# Basement membrane and vascular remodelling in smokers and chronic obstructive pulmonary disease: a cross-sectional study

**DOI:** 10.1186/1465-9921-11-105

**Published:** 2010-07-30

**Authors:** Amir Soltani, David W Reid, Sukhwinder S Sohal, Richard Wood-Baker, Steve Weston, H Konrad Muller, E Haydn Walters

**Affiliations:** 1 Respiratory Research Group, Menzies Research Institute, University of Tasmania, 17 Liverpool St, Hobart, 7000, Australia; 2 Discipline of Pathology, Menzies Research Institute, University of Tasmania, 17 Liverpool St., Hobart, 7000, Australia

## Abstract

**Background:**

Little is known about airway remodelling in bronchial biopsies (BB) in smokers and chronic obstructive pulmonary disease (COPD). We conducted an initial pilot study comparing BB from COPD patients with nonsmoking controls. This pilot study suggested the presence of reticular basement membrane (Rbm) fragmentation and altered vessel distribution in COPD.

**Methods:**

To determine whether Rbm fragmentation and altered vessel distribution in BB were specific for COPD we designed a cross-sectional study and stained BB from 19 current smokers and 14 ex-smokers with mild to moderate COPD and compared these to 15 current smokers with normal lung function and 17 healthy and nonsmoking subjects.

**Results:**

Thickness of the Rbm was not significantly different between groups; although in COPD this parameter was quite variable. The Rbm showed fragmentation and splitting in both current smoking groups and ex-smoker COPD compared with healthy nonsmokers (p < 0.02); smoking and COPD seemed to have additive effects. Rbm fragmentation correlated with smoking history in COPD but not with age. There were more vessels in the Rbm and fewer vessels in the lamina propria in current smokers compared to healthy nonsmokers (p < 0.05). The number of vessels staining for vascular endothelial growth factor (VEGF) in the Rbm was higher in both current smoker groups and ex-smoker COPD compared to healthy nonsmokers (p < 0.004). In current smoker COPD VEGF vessel staining correlated with FEV1% predicted (r = 0.61, p < 0.02).

**Conclusions:**

Airway remodelling in smokers and mild to moderate COPD is associated with fragmentation of the Rbm and altered distribution of vessels in the airway wall. Rbm fragmentation was also present to as great an extent in ex-smokers with COPD. These characteristics may have potential physiological consequences.

## Background

Chronic obstructive pulmonary disease (COPD) and asthma are both common chronic inflammatory respiratory diseases. COPD is a world wide problem mainly caused by cigarette smoking. Although COPD is the fourth most common cause of chronic disability and mortality in developed countries and its prevalence is increasing, little is known about structural, or remodelling, changes in the airway wall and their relation to physiology [1,2]. This contrasts with the wealth of available data in asthma where alterations in the reticular basement membrane (Rbm) (lamina reticularis) morphology and subepithelial tissue hypervascularity are acknowledged as important features of airway wall remodelling [3-5]. Furthermore, there have been few reports on larger airway structural changes using bronchial biopsies (BB) in COPD, with most published data relating to the lung parenchyma and small airways in surgically resected specimens [4].

Given that COPD is also a chronic inflammatory pan-airway disease we initially decided to study BB from COPD subjects and compare them with nonsmoking healthy volunteers. This preliminary study revealed Rbm and vessel changes in BB from COPD subjects that have not been previously reported. The Rbm was nonhomogenous in appearance and fragmented. Fragmentation included cleft formation and splitting within the Rbm. Vessels found in contact, penetrating and indeed completely within the Rbm. On the basis of these findings we hypothesised that Rbm splitting and Rbm vascularisation are specific features of COPD. We also hypothesised that these changes may, like in asthma, [6] be related to VEGF activity.

In this paper we report our findings on Rbm and vascular remodelling in BB from COPD patients (current-smokers and ex-smokers) and compare them with both healthy smokers and healthy nonsmokers.

## Methods

### Study design

This was a cross-sectional study.

### Subjects

We recruited 65 subjects through advertisement. To test our hypotheses, we have compared BB from 17 healthy, nonsmoking subjects (H-N), 19 current smokers with COPD (S-COPD) and 14 ex-smokers with COPD (ES-COPD, all had quit for at least 6 months). To further discriminate between smoking and disease effects we included BB from 15 current smokers with normal lung function (S-N).

COPD was diagnosed according to the GOLD guidelines [7]. Volunteers with a history of other lung diseases were excluded. Subjects who reported exacerbations or systemic or inhalational corticosteroid use during last 12 weeks were excluded. Patients were on symptomatic treatment with anticholinergics when recruited and during the study.

The study was *approved by the Human Research Ethics Committee (Tasmania) Network*. All subjects provided written informed consent.

*Pulmonary function tests *were performed according to the ERS/ATS guidelines [8].

*Bronchoscopies *were performed as previously described [5]. Eight BB from the secondary carina of segmental and subsegmental bronchi were obtained. There were no complications from the procedures.

### Tissue processing

4 biopsies were collected in saline of which 2 were subsequently snap frozen in liquid nitrogen/isopentane slurry and embedded in OCT for possible immunostaining and the other 2 in liquid nitrogen for molecular analysis at a later date. All 4 were stored at -80°C. The other 4 biopsies were fixed in 4% neutral buffered formalin for 2 hours and subsequently processed into paraffin through graded alcohol and xylene using a Leica ASP 200 tissue processor. Sections were cut at 3 microns from individual paraffin blocks, stained with Haematoxylin and Eosin and morphologically assessed for immunostaining. Blocks stained were chosen to minimize tangential sectioning of the epithelium and to provide greatest length of epithelium for assessment. Two 3 micron sections from appropriate blocks were collected on each slide being separated by a minimum of 50 microns. Following removal of paraffin and hydration to water immunostaining for Collagen-IV (Dakocytomation, Denmark, cat. no. M0785 clone CIV 22: 1/100 dilution, 90 minutes at room temp with heat retrieval) and Vascular Endothelial Growth Factor (VEGF) (Fitzgerald, Concord MA. Cat. no. 10R -V101ax: 1/500 dilution, overnight at room temperature) was performed on separate slides. In each case a non immune IgG1 negative control (Dakocytomation, Denmark X0931 clone DAK-GO1) was performed to eliminate false positive staining. Bound antibodies were elaborated using Peroxidase labeled Envision + (Dakocytomation, Denmark cat. no. K4001) and liquid DAB + (Dakocytomation, Denmark cat. no. K3468).

### Measurements

Sections were randomized by author SW independently of the person who examined them (AS) that was blinded to diagnosis and order. Tissue examination was performed using computer assisted image analysis tool (Image-Pro version 5.1, Media Cybernetics, USA) at x400 magnification. As many pictures as possible were taken from each slide. Then 8 separate fields were randomly chosen for examination.

Thickness of the Rbm was assessed by first identifying the outer subepithelial border of the true basement membrane and then the inner border of Rbm. The average distance between these two borders along the length of the Rbm within the microscopic field of vision was then measured with the aid of automated software. 3 mm length of the Rbm was included in the measurement.

**Figure 1:**
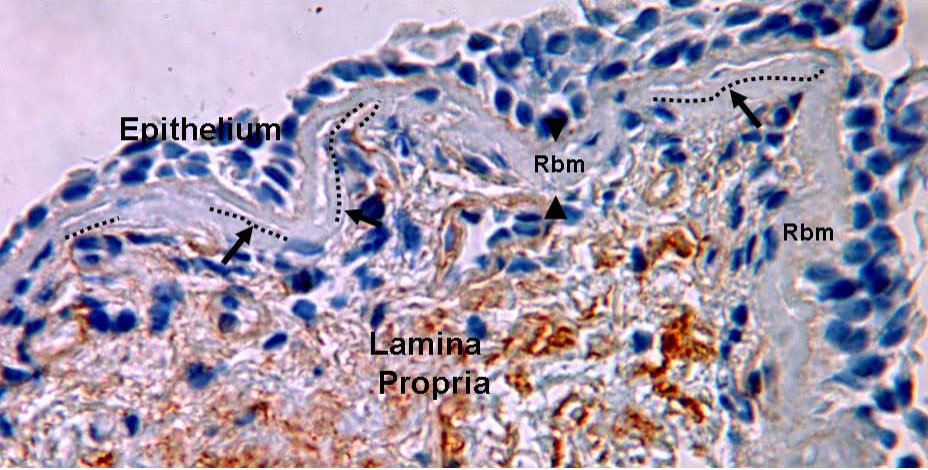
**Rbm splitting**. Splits within the lamina reticularis (Rbm) are indicated by black arrows. The dotted lines are examples of how we measured splits. The borders of the Rbm are marked by arrow-heads. (Collagen IV antibody staining, × 400).

**Figure 2:**
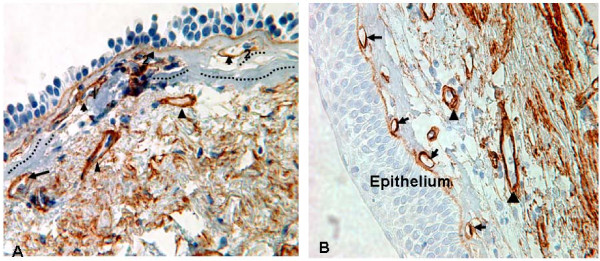
**Rbm vessels**. **A**. Rbm-associated vessels (indicated by black arrows) are in close contact with, are penetrating (1) or are embedded within (2) the Rbm. Arrow-heads point to vessels in the LP. Rbm splits are indicated again by dotted lines. **B**. Rbm-associated vessels (indicated by black arrows) are embedded within the Rbm. Arrow-heads point to vessels in the LP. (Collagen IV staining, × 400).

Fragmentation of the Rbm included pieces apparently hanging off and indeed completely separated from the remainder, but also was associated with splitting and formation of clefts within the Rbm (Figures 1 &2). We have used this splitting as a quantitative measure for the observation. The total length of splits was summated and divided by the length of Rbm. Where the splits were in parallel layers, all of them were included in the measurement.

Rbm-associated vessels included those in contact with the inner surface of the Rbm, penetrating it, or embedded within it (Figure 2). "Vascular area" was assessed as the area enclosed by the Collagen IV staining of the endothelial basement membrane. The area of all vessels was measured using the image analysis tool and then added together. Measurements were normalised by dividing by the length of Rbm.

The number and area of vessels within the LP (Figure 2) were counted to a depth of 150 micrometer from the internal border of Rbm. Those vessels counted as Rbm vessels were excluded. Vascular density and %vascular area of the LP were calculated by dividing the total number of vessels and vascular area by the total surface area of the LP examined.

The vessels and cells stained with VEGF in the Rbm and LP were also quantified.

Sixty three subjects had enough tissue for assessment of VEGF and fifty nine for Collagen IV staining.

### Analyses

Non-parametric ANOVA (Kruskal Wallis) and post hoc Mann-Whitney U tests were used for testing mean differences in variables with non-normal distribution. For normally distributed variables ANOVA and post hoc t tests were used. Spearman and Pearson correlation analyses were used as indicated to test relationships. All analyses were performed by SPSS 15 for windows. Two-tailed p values < 0.05 were considered as significant.

*Repeatability *of our measurements was tested by blinded re-examination of 12 randomly selected slides and calculating the coefficient of repeatability for our indices of interest by the method of Bland and Altman [9]. This indicates what degree of change one can pick significantly up over time or with an intervention. For the outcomes reported here, the mean differences between paired counts were very close to zero and we obtained coefficients of repeatability of 33 to 94% of the mean counts, which are comparable to or better than previous analyses of this kind in airway biopsy material [10]. We also tested reliability by intra-class correlation coefficients (using ICC 3,1), which varied from 0.83 to 0.97 and therefore are in the satisfactory range [11].

## Results

*Demographics *of participants are presented in Table 1. COPD subjects were significantly older than the others and by definition had lower FEV1% predicted, FEV1/FVC ratio and diffusion of carbon monoxide (DLCO) % predicted (p < 0.01).

**Table 1:** Demographics of study group

Groups* (numbers)	H-N (17)	S-N (15)	S-COPD (19)	ES-COPD (14)
Age^† ^years	49 (20-68)	46 (30-65)	61 (46-78)	62 (53-69)
Female/Male	11/6	4/11	8/11	5/9
Pack-Years smoking^†^	0	35 (11-57)	45 (18-82)	55 (18-151)
FEV1% Predicted^†‡^	119 (114-124)	100 (78-125)	83 (55-102)	83 (55-105)
FEV1/FVC% ratio^†‡^	82 (71-88)	78 (70-96)	59 (46-68)	57 (38-68)
DLCO% predicted^† ^ml/min/mmHg	-	77 (58-105)	67 (48-83)	64 (45-74)
Number (%) with DLCO% Predicted < 60%	-	1 (6.7%)	2 (11.2%)	4 (30.8%)

*H-N: healthy and nonsmoker, S-N: smokers with normal lung function, S-COPD and ES-COPD: smoker and ex-smoker COPD

† Median (range), ^‡ ^post-bronchodilator values

### Rbm morphology (Figure 3)

**Figure 3:**
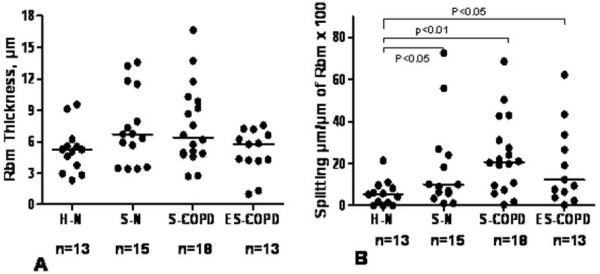
**Rbm thickness and splitting compared between groups**. **A**. Rbm thickness was not different between groups (p = 0.13). **B**. Compares length of splitting between groups. Bars indicate medians.

Thickness of the Rbm was not significantly different between the four groups (p = 0.13) but was especially variable in COPD subjects. The total length of Rbm splits (Figures 1, 2 &3) in S-N and both COPD groups was significantly greater than in normal controls (p < 0.02). Splits were not significantly different between S-N, S-COPD and ES-COPD groups. Numerically, splitting was greatest in S-COPD, but this did not reach a conventional level of significance.

### Vessels in the Rbm (Figure 4)

Both the number and area of vessels in the Rbm (Figure 2) were significantly different in current smoking groups compared to H-N (p < 0.05) while in the ES-COPD group vascularity was essentially normal. The area of vessels was significantly higher in S-N than ES-COPD and when both current smoking groups were compared with ES-COPD (p < 0.05).

**Figure 4:**
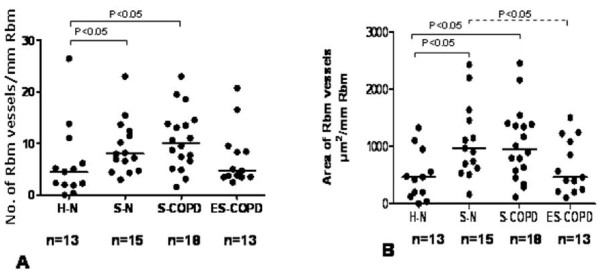
**A & B. Current smokers have more vessels and larger vascular area in the Rbm**. Bars indicate medians.

### Vessels in the LP (Figure 5)

The density of vessels in the LP was significantly lower in the two currently smoking groups compared to H-N (p < 0.005), while ES-COPD had normal values. S-N and both current smoking groups together had significantly lower vascular density than ES-COPD (p < 0.01 and p < 0.02).

**Figure 5:**
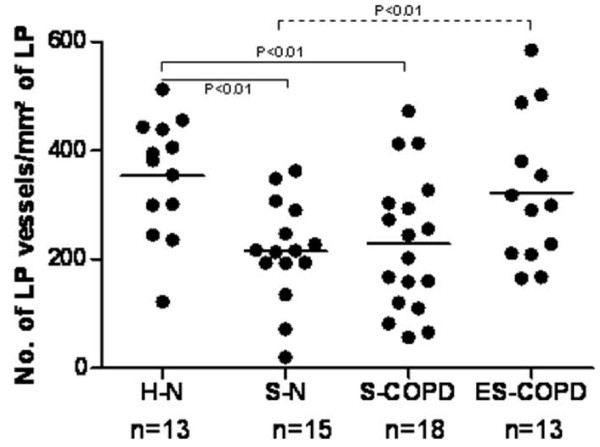
**There are fewer vessels in the LP in current smokers**. Bars indicate means.

### VEGF (Figure 6)

The number and area of vessels stained with VEGF in the Rbm were significantly different between groups (p < 0.004), the increase being most marked for the S-COPD group. The proportion of vessels stained with VEGF (ratio of vessels stained with VEGF divided by total number of vessels) in the Rbm was significantly higher in S-N, S-COPD and ES-COPD compared to H-N (p < 0.006) (data not shown). There were no differences between groups in cells or vessels stained for VEGF in the LP, nor in VEGF-stained cells in the Rbm.

**Figure 6:**
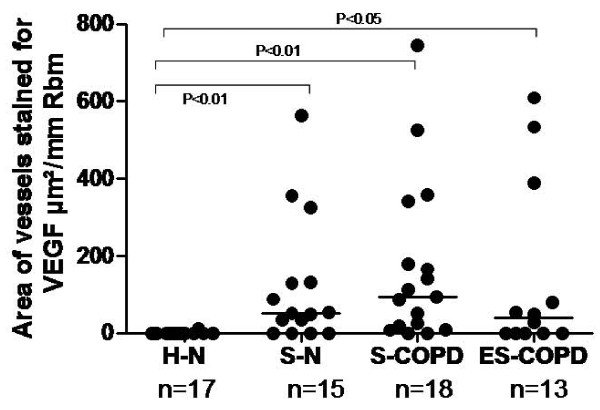
**Vessels stained for VEGF are compared between groups**. Bars indicate medians.

### Correlations

**Figure 7:**
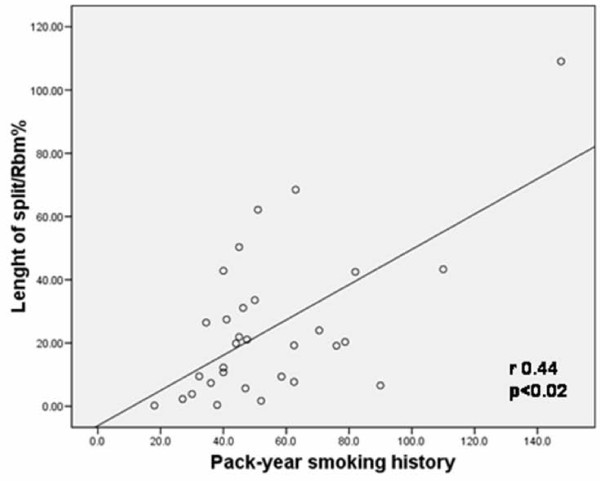
**Length of splitting is related to pack-year smoking history in both COPD groups taken together**.

Pack-year history of smoking and splitting of the Rbm were positively correlated (r = 0.44, p < 0.02) for the two COPD groups (Figure 7). S-COPD group showed a positive correlation between FEV1% predicted and vessels positive for VEGF in the Rbm (r = 0.61, p < 0.02) and also a positive correlation between vessel number in the LP and FVC% predicted (r = 0.5, p < 0.05). We did not find any suggestion of a relationship between age and any of the pathological findings in any group or combination of groups.

## Discussion

This study has revealed new aspects of airway remodelling in the large airways in smokers with or without COPD. We have attempted to differentiate effects of smoking from the presence of established disease as defined by the GOLD initiative.

Our main results may be summarised as follows:

1. Rbm thickness was not different between groups.

2. The Rbm was fragmented and had markedly increased splitting in smokers and COPD (Figures 1 and 2), and especially in current smoking COPD.

3. The Rbm was hypervascular in smokers but not in ES-COPD.

4. The LP was hypovascular in smokers but not in ES-COPD.

5. Vessel staining for VEGF was increased in smokers and COPD, but especially in current smokers with COPD.

We did not find a significant difference between groups in Rbm thickness. Previous studies have been contradictory. One group found thicker Rbm in COPD compared with controls, [12] with both COPD and control groups in this study being ex-smokers except for 3 COPD subjects who were never smokers. Others have not found this difference [13,14]. We did find the Rbm thickness to be very variable in smokers and in COPD, and because of the fragmentation it was less easy to quantitate accurately.

The main changes in the Rbm in smokers and COPD were marked fragmentation and hyper-vascularisation which are novel findings and not previously published in the COPD literature. Rbm splitting, we propose, could be the result of either new layers being formed by the epithelium or more likely degradation of the Rbm by proteolytic enzymes. Rbm splitting has been reported previously in the glomerular basement membrane and endothelial basement membrane of tubules in kidney transplant rejection [15,16]. Cornell *et al. *proposed that splitting is the consequence of repeated episodes of injury with new basement membrane layers formed as part of a repair process.

Smoking induces repeated injury to the airway epithelium. As Cornell *et al. *proposed for kidney rejection, this may induce epithelial repair with formation of a new layer in the Rbm. This is compatible with the correlation of smoking history and length of splitting in our study and also explains the observed nonhomogeneity of the width of the Rbm in smokers.

However, the presence of splitting may well represent a change or degradation in Rbm matrix proteins. We believe the changes are unlikely to be an artifact of processing as this was the same for all groups, and in previous work in asthma, where those changes are not seen. Recently, differences in the components of collagen and other proteins in the Rbm in a study comparing asthma, COPD and controls have been described [12]. Change in proteinase activity, which has been shown in COPD, [17] may potentially explain this phenomenon. The correlation of splitting with historical amounts of smoking confirms that it is likely related to cumulative insult to the airway mucosa.

Although COPD subjects were significantly older than the control group, there was no correlation between age and the length of splitting in either COPD group, analysed separately or together, nor in the S-N group. Multivariable analysis showed that age is not a predictor of splitting (p = 0.4) but pack-year smoking history is (p < 0.02) (Table 2). The presence of splitting in ES-COPD means we need a longitudinal study to assess whether the Rbm is truly unable to repair itself after smoking cessation, and to relate this to proteinase activity.

**Table 2:** Correlation analysis for Rbm splitting for both COPD groups and smokers with normal lung function*^†^

	Regression Coefficient	95% CI	P value
Age (years)	+0.36	-0.50 to +1.22	0.4
Pack-year smoking	+0.43	+0.10 to +0.76	<0.02

*CI = Confidence interval

^†^The regressions are also adjusted for gender

Current smokers, irrespective of their pulmonary function, had increased vessel numbers in relation to the Rbm. This pathological change may be reversible with smoking cessation, as ES-COPD was not different from H-N but was different from S-N and both current smoker groups taken together. Again a longitudinal smoking cessation is now needed to confirm this and explore the mechanisms involved. We stained a number of matched slides with Factor VIII, which stains endothelium of blood vessels [18], which confirmed that the structures stained by Collagen IV were indeed vessels.

We found more vessels stained for VEGF in the Rbm of current smokers and COPD, but VEGF staining was most marked in current smoking COPD subjects. VEGF is present in actively proliferating endothelium and is a marker of active angiogenesis [19]. Therefore, we suggest that angiogenesis appeared to be equally active in COPD subjects who had quit smoking, suggesting that it is not reversible. Again, a properly designed longitudinal smoking cessation study will be necessary to confirm this.

In contrast, we found fewer blood vessels in the LP in current smokers, but not in ES-COPD. There have been few previous studies investigating vascular changes in large airway endobronchial biopsies in COPD, and none to our knowledge that have differentiated between the Rbm and LP. Calabrese *et al. *in a study on bronchoscopically-obtained biopsies reported more vessels in the LP of smokers, and concluded that angiogenesis is a part of airway remodelling in smokers. They did not find any relationship between remodelling changes and lung function or clinical manifestations [20]. Another recent Italian study found larger vascular area in BB from ex-smokers with moderate to severe COPD compared to control subjects. The number of vessels was not different between groups [21].

A potential explanation for these previous findings, which appear to contrast with our own, would be the different selection criteria employed. For example, Calabrese *et al. *recruited smokers with normal lung function or COPD with clinical criteria of chronic bronchitis and they excluded subjects with emphysema. Chronic bronchitis, which at least anecdotally is not as prominent a feature of COPD in Australia as in Europe, was almost completely absent in our S-N subjects without being selected on this basis. We did not exclude subjects with emphysema in our COPD groups (Table 1) and tried to include a "typical" local COPD population. Zanini *et al. *recruited moderate to severe COPD subjects that had quit for more than 10 years and they did not study current smoker COPD subjects. We studied COPD subjects with mild to moderate COPD. In our study current smokers with COPD had the most marked changes. Further, we separately counted vessels in the Rbm and LP. However, if the Rbm- associated vessels were added to vessels in the LP we still found fewer vessels overall in the mucosa in current smokers (data not shown).

There are other studies that examined airway vascularity in COPD but used subjects with peripheral lung cancer to study only smaller airways in lung resection specimens [22,23]. Hashimoto *et al. *did not find any differences in vessels in medium sized airways (internal diameter 2-5 mm) between COPD and nonsmoking controls, and Kuwano *et al. *did not find a significant difference in vessel density in the mucosa of peripheral airways in subjects with mild COPD compared with controls without airway disease.

The reason for hypovascularity of the LP in smokers in our study could not be explained on the basis of the VEGF data produced. Pulmonary VEGF reduction in smokers has been reported [24,25]. Hypovascularity of the LP in current smokers may be analogous to the observation that down-regulated VEGF within the lung parenchyma is associated with the development of emphysema [26,27]. Our current study did not find reduced VEGF activity either in the Rbm or in the LP in current smoker groups, with the percentage of vessels in the LP staining for VEGF not being significantly different between groups. However, an explanation for this apparent paradox could be that VEGF is functionally unavailable for new vessel formation in the presence of cigarette smoke [28]. The finding of normal vessels in ES-COPD supports this idea. More studies of the angiopoietic system in the airways in smokers are indicated. Deprivation of other angiogenic factors, such as angiopoietin-1 and/or down-regulation of endothelial VEGF receptors should also be considered and studied. Whatever the mechanism, hypovascularity of the LP is a smoking effect that may be reversible with quitting, but a specific longitudinal study is needed to confirm that.

The strong relationship between Rbm vessel-related VEGF and better FEV1% predicted in S-COPD group is interesting. There is some evidence that some aspects of remodelling may have a protective effect [2,29,30], and potentially angiogenesis in the Rbm could increase airway stiffness and resist dynamic compression which is frequently a physiological problem in COPD. Similarly, the positive correlation between FVC% predicted and the number of vessels in the LP in the S-COPD group, probably reflecting less air trapping with more LP vessels, supports this idea. However, this is likely to be a reflection of the situation in the small airways which were not sampled in our study. These suggestions could be confirmed by direct assessment of airway distensibility in future studies [31]. Thus, at this stage we can not confirm that the associations between vessel changes and lung function are causative and further investigation is required.

COPD groups in our study were significantly older than H-N and S-N. However, the age range in COPD was wide and detailed uni- and multi-variable analyses did not suggest that age was a factor influencing the main findings.

## Conclusions

This study examined novel aspects of Rbm and vascular remodelling in large airway biopsies in current smokers with normal lung function and patients with established mild to moderate COPD. Most changes seemed related to smoking, but some were most marked in current smoking COPD patients, suggesting additive effects in this situation. Vessel changes may be reversible with quitting but not Rbm fragmentation. Vascular changes in the Rbm and LP were in opposite directions in current smoking groups; the Rbm was hypervascular and the LP was hypovascular. Hypervascularity of the Rbm was associated with increased VEGF expression that was positively related to better lung function in current smokers with COPD. Further investigations are needed to study the VEGF system and receptors in greater depth, and other angiogenic factors that may contribute to vascular remodelling and redistribution in the airways of smokers with or without COPD. Longitudinal studies to confirm the effects of smoking cessation and assess disease-modifying therapy such as inhaled corticosteroids on airway remodelling in COPD are needed to help clarify the pathophysiological significance of our findings.

## List of abbreviations

BB: bronchial biopsies; COPD: chronic obstructive pulmonary disease; DLCO: diffusion of carbon monoxide in the lung; ES-COPD: Ex-smokers with COPD; FEV1: Forced expiratory volume in first second; FVC: Forced vital capacity; H-N: Healthy and never-smoking; LP: Lamina propria; Rbm: Reticular basement membrane (lamina reticularis; a layer attached to and beneath the true basement membrane); S-COPD: Current smokers with COPD; S-N: Smokers with normal lung function; VEGF: Vascular endothelial growth factor

## Competing interests

The authors declare that they have no competing interests

## Authors' contributions

AS has written the first draft of this manuscript. SSS and SW contributed to the writing of this manuscript. EHW, RWB and DWR designed the study and supervised the research and the writing of this paper. HKM supervised the study and advised on the histopathological aspects of the paper. This manuscript has been read and approved by all authors.
